# First Identification of Resident and Circulating Fibrocytes in Dupuytren’s Disease Shown to Be Inhibited by Serum Amyloid P and Xiapex

**DOI:** 10.1371/journal.pone.0099967

**Published:** 2014-06-16

**Authors:** Syed Amir Iqbal, Michael John Hayton, James Stewart Watson, Piotr Szczypa, Ardeshir Bayat

**Affiliations:** 1 Plastic & Reconstructive Surgery Research, Manchester Institute of Biotechnology, University of Manchester, Manchester, United Kingdom; 2 Department of Hand and Upper Limb Surgery, Wrightington Hospital, Wrightington, United Kingdom; 3 Department of Plastic and Reconstructive Surgery, University Hospital South Manchester NHS Foundation Trust, Wythenshawe Hospital, Manchester, United Kingdom; 4 Medical Affairs Pfizer Ltd, Tadworth, Surrey, United Kingdom; University Medical Center Freiburg, Germany

## Abstract

Dupuytren’s disease (DD) is a common progressive fibroproliferative disorder causing permanent digital contracture. Proliferative myofibroblasts are thought to be the cells responsible for DD initiation and recurrence, although their source remains unknown. DD tissue has also been shown to harbor mesenchymal and hematopoietic stem cells. Fibrocytes are circulating cells that show characteristics of fibroblasts and they express surface markers for both hematopoietic and mesenchymal stromal cells. Fibrocytes differentiate from peripheral CD14^+^ mononuclear cells, which can be inhibited by serum amyloid P (SAP). In this study we have demonstrated the presence of fibrocytes in DD blood and tissue, moreover we have evaluated the effects of SAP and Xiapex (Collagenase *Clostridium histolyticum*) on fibrocytes derived from DD. H&E staining showed typical Spindle shaped morphology of fibrocytes. FACS analysis based on a unique combination of 3 markers, revealed the increased presence of fibrocytes in blood and tissue of DD patients. Additionally, immunohistology of DD nodule and cord tissue showed the presence of collagen 1^+^/CD34^+^ cells. No difference in plasma SAP levels was observed between DD and control. Higher concentrations of SAP significantly inhibited fibrocytes differentiated from DD derived monocytes compared to control. DD fascia derived fibrocytes showed resistance to growth inhibition by SAP, particularly nodule derived fibrocytes showed robust growth even at higher SAP concentrations compared to control. DD derived fibrocytes were positive for typical fibrocyte dual markers, i.e. Collagen 1/LSP-1 and collagen 1/CD34. Xiapex was more effective in inhibiting the growth of nodule derived cells compared to commercially available collagenase A. Our results show for the first time the increased presence of fibrocytes in DD patient’s blood and disease tissue compared to control tissue. Additionally, we evaluate the response of these fibrocytes to SAP and Xiapex therapy.

## Introduction

Dupuytren’s disease (DD) is a common fibroproliferative disorder of unknown etiology that is characterized by progressive and irreducible digital flexion contractures [1]. The disease affects more than 7% of United States population and around 25% of men older than 60 years of age of Northern European descent [2], [3]. DD often starts with a small painless nodule in the connective tissue (fascia) of the palm that eventually develops into a cord-like band that prevents full extension of the finger [4]. The current treatment for DD includes surgical fasciectomy, percutaneous needle fasciectomy and Xiapex, a form of Collagenase *Clostridium histolyticum* (CCH) injection [5]. Notwithstanding various treatment options, fasciectomy remains a common surgical procedure for management of DD. Although familial heritability and DD diathesis have been attributed to a higher disease recurrence, nevertheless, the exact etiology leading to this elusive condition remains unknown [6]. It is possible that disease recurrence is caused by a resident or circulating source of abnormal progenitor cells that are capable of differentiating into disease specific fibroblasts/myofibroblasts [7].

Myofibroblasts are considered to be responsible for development and progression of DD pathogenesis and even recurrence is thought to be associated with re-appearance of myofibroblasts [8], [9]. Previously, DD cord and nodule have been shown to harbor progenitor cells including a population of mesenchymal stem cells (MSCs) thought to be a primary source of myofibroblasts [10]–[13]. Therefore, the recruitment of progenitor cells from surrounding areas and their differentiation into myofibroblasts is a likely scenario although not peculiar to DD. For instance, hepatic stellate cells differentiate to myofibroblasts in the liver and epithelial-mesenchymal transition provides another mechanism to generate myofibroblasts in pulmonary and renal fibrosis [14]–[16]. In addition, it is suggested that fibrocytes that circulate in the blood can migrate to specific tissues and differentiate into myofibroblasts as shown previously in renal fibrosis [16].

Fibrocytes are derived from peripheral blood mononuclear cells (PBMCs) and exhibit features of both hematopoietic and stromal cells [17]. Fibrocytes are identified on the basis of expression of unique combinations of markers that include CD34, CD45, major histocompatibility complex II, lymphocyte-specific protein 1 (LSP-1) and collagen I [18]–[20]. After injury fibrocytes transform into myofibroblasts with increased production of collagen at the wound site [21]. Fibrocytes, in addition to acting as myofibroblasts precursors, also secrete a variety of cytokines, growth factors, and other extracellular matrix proteins (ECM) which are shown to be up-regulated in DD tissue such as transforming growth factor (TGF)-β, tumor necrosis factor (TNF)-α, interleukin (IL)-6, vascular endothelial growth factor (VEGF), platelet derived growth factor (PDGF), type I collagen, a-smooth muscle actin, and fibronectin [22].

In previous studies, serum-enriched media was found to inhibit fibrocyte differentiation in culture. The inhibitory component in serum was found to be serum amyloid P (SAP), a stable serum protein later shown to slow the rate of dermal wound healing, presumably by inhibiting fibrocytes differentiation at the wound site [23], [24]. Fibrocytes have been previously studied in hypertrophic scarring; another form of fibroproliferative dermal wound healing. Yang et al. showed a twofold increase in fibrocytes in the hypertrophic scar of burn patients compared with normal skin and mature skin scars, and further work revealed the number of fibrocytes correlated with burn severity [25]–[27]. We have recently shown the presence of fibrocytes in keloid disease tissue on the basis of combination of inflammatory markers [28]. We have selected CD45RO, 25F9 and MRP8/14 based on a previous study that showed (from immunohistochemistry alone) that fibrocytes could be uniquely identified when all three markers are expressed simultaneously [29]. 25F9 and MRP8/14 are expressed on macrophages alone while CD45RO is expressed on leukocytes. With the secretory profile of fibrocytes mirroring the composition of the DD milieu, and the known link between fibrocytes and hypertrophic scarring, we hypothesized that the peripheral blood mononuclear cells (PBMNCs) and cells derived from DD tissue would differentiate more readily into fibrocytes *in vitro* when compared to control subjects and that SAP can be used to inhibit this differentiation.

In addition to SAP we also investigated the effects of Xiapex (Auxilium Pharmaceuticals, USA, trade name Xiaflex in USA), a specific form of collagenase that is a combination of collagenases AUX-I:AUX-II from *Clostridium histolyticum* [30]. The effects of collagenase on the collagenous components of DD cords (with morphologic sparing of cellular elements) have been well described in *in vitro* explant cultures [31]. However, there has been little published data to date regarding the functional effects of CCH on DD tissue at a cellular level. We recently showed the effects of Xiapex on DD fibroblasts and demonstrated that Xiapex significantly down-regulated the formation of ECM components compared to control fascial fibroblasts [32].

Thus, the main aim of this study was to identify fibrocytes derived from DD blood and tissue. In addition, we investigated the functional effects of SAP, Xiapex and commercially available collagenase A (Roche, UK) on cells cultured from DD nodule, DD cord, surrounding fat (used as internal control), and overlying skin (mainly dermis), as well as on mononuclear cells derived from DD and control fascia.

## Materials and Methods

### Patient Data

This study was carried out in accordance with the Declaration of Helsinki and was approved by the NHS national research ethics committee in England, UK. Blood and tissue were collected after patients had given informed written consent prior to their surgical procedure (Table 1 for DD patient data). The blood and tissue were handled and disposed of according to the guidelines provided by UK human tissue act (HTA).

**Table 1:** Demographic Data of Dupuytren’s Disease Cases and Control Carpal Tunnel Subjects.

Description	Patients with Dupuytren’s Disease	Controls subjects undergoing Carpal Tunnel Release
Total Patient Count	20	10
Gender (Male/Female)	15/5	8/2
Race or Ethnicity	Caucasian	Caucasian
Age in Years, Mean	67	56
Age Range, in Years	42–85	38–69
Positive Family History of Dupuytren’s Disease (%)	15 (50)	0 (0)
Varying Severity of Disease, Severity Score	7–10	NA

### Collection of Blood and Tissue Samples

**Figure 1:**
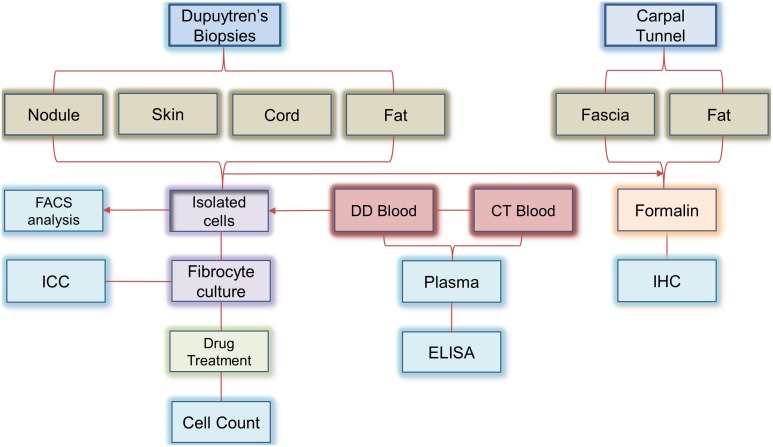
A flow chart depicting the study design. The flow chart here describes how the obtained tissue samples that were utilized for confirmation of the presence of fibrocytes in Dupuytren’s Disease.

Twenty ml of blood was collected from DD (n = 10) and control carpal tunnel (CT) (n = 10) patients in sodium heparin vacutainers (BD Bioscience, California, USA). Biopsies of DD cord, nodule, fat (used as an internal control) and skin were obtained from patients undergoing routine fasciectomy surgery for Dupuytren’s disease (n = 20, age range; 42–85 yrs., mean age = 67 yrs., 15 males and 5 females, Table 1). Control subjects tissue (n = 5) were individuals without personal or family history of DD who were undergoing routine, non-neoplastic surgical procedure on the palm of their hand (all control subjects underwent routine surgical release of their carpal tunnel for treatment of carpal tunnel disease). We were unable to use palmar fascia from patients undergoing CT procedure as an external control as the number of cells obtained from individual patient’s biopsy was approximately 20–30×10^4^ cells, which were insufficient for performing a drug assay. All samples obtained were placed in Dulbecco’s minimal essential medium (DMEM; PAA, UK) and in 10% neutral buffered formalin (Sigma-Aldrich, UK). Figure 1 explains the overall plan of this study.

### Fluorescence Activated Cell Sorting (FACS)

Fibrocytes were identified using primary antibodies (all diluted 1∶20 in Hank’s balanced salt solution (HBSS), PAA, UK) against CD45RO (conjugated with phycoerythrin, PE, [BD Bioscience, UK]), 25F9 (conjugated with Alexa Fluor 647, AF647, [eBioscience, UK]) and MRP8/14 (conjugated with fluorescein isothiocyanate, FITC, [eBioscience, UK]). Samples were washed once with HBSS and centrifuged down into a pellet. Samples were then incubated in 100 µl diluted primary antibody cocktail on ice for 30 minutes. Cells were washed once with HBSS and resuspended in the same buffer in 500 µl HBSS for FACS analysis. 7AAD (BD Bioscience, UK) was added to determine cell viability. To assess non-specific binding, isotype-matched antibodies (eBioscience, UK) conjugated to corresponding fluorophores used in primary antibodies were used. Data were acquired on Accuri C6 (BD Bioscience, USA).

### Serum Amyloid P Detection by ELISA

Plasma was obtained from whole blood of DD cases (n = 5), and control CT subjects (n = 6) within 2 hrs of collection by centrifuging blood at 1500 *g* for 10 min. Collected plasma was further centrifuged for 10 min at 2000 *g* to get rid of any platelets and red blood cells contamination. SAP concentration in plasma was detected using Hycult SAP ELISA detection kit (Hycult Biotech, The Netherlands) according to the supplier’s instructions. Briefly plasma was diluted 1∶1000 in diluting buffer provided and 100 µl of it was added to the plate already coated with SAP capturing antibody. After incubation for an hour at room temperature (RT) the plate was washed 4x with washing buffer provided. 100 µl of biotinylated anti-SAP antibody was added to the mixture and incubated again for an hour at RT. After washing 4x with washing buffer, 100 µl of diluted streptavidin-peroxidase was added to the plate and incubated at RT for an hour. After another wash step 100 µl of 3,3′,5,5′–tetramethylbenzidine (TMB) was added and incubated at RT for 30 min. The reaction was stopped using the provided stop buffer and the plate was read at 450 nm. A standard curve of known SAP concentrations (0.2 to 50 ng/ml) was plotted vs. optical density (OD) values and unknown SAP concentrations of samples were read using interpolation.

### Fibrocyte Culture from DD Tissue

Tissue biopsies were processed to obtain fresh cells as explained earlier [7]. Briefly, tissue was washed once with phosphate buffered saline (PBS; PAA, UK) before being minced and incubated in collagenase A (5 mg/ml, Roche Diagnostics, UK) at 37°C for 16 hrs. The cell suspension was then passed through a 70 µm cell strainer (BD Biosciences, USA) to remove any remaining tissue. Cells were then washed once with PBS and then passed through a nylon mesh filter (50 µm Filcon, BD Biosciences, USA) to be used immediately as fresh cells for FACS analysis and cell culture. Cells were maintained in culture up to 8 days in FibroLife medium (CellSystems Biotechnologie Vertrieb GmbH, Germany) supplemented with 2 mM L-glutamine, 100-units/ml penicillin, 100 mg/ml streptomycin and 1x ITS+3 (Sigma-Aldrich, UK). Cells were grown in 96-well plate (Corning, USA) and kept at 37°C/5% CO_2_. For immunofluorescence cells were also grown in 8-well chamber slides (LabTek, Sigma-Aldrich, UK).

### Fibrocyte Culture from CT and DD Blood

Peripheral blood samples were process within 8 hrs of retrieval and mononuclear cells (MNCs) extracted and cultured. The blood was mixed 1∶1 with 1x sterile PBS and MNCs were isolated using Ficoll-Paque Plus (GE Healthcare, VWR, UK). Isolated MNCs were re-suspended in serum-free FibroLife Media (Lifeline Cell Technology, Walkersville, MD) supplemented with 10 mM HEPES solution (Sigma-Aldrich), 1x nonessential amino acids (Sigma-Aldrich), 2 mM L-glutamine (Sigma-Aldrich), 1 mM sodium pyruvate (Sigma-Aldrich), 100-units/mL penicillin (Sigma-Aldrich), 100 mg/mL streptomycin (Sigma-Aldrich), and 1X insulin-transferrin-sodium selenite (ITS) solution (Sigma-Aldrich). PBMCs were cultured in 96-well flat bottom culture plates (Corning, Corning, NY) in 200 µL with increasing concentrations of human SAP (Sigma-Aldrich) from 0 to 10 µg/ml and with increasing concentrations of Xiapex (provided by Pfizer, UK) from 0 to 40 µg/ml. Each set of PBMCs was cultured in duplicate and incubated at 37°C with 5% CO_2_.

### Enumeration of Cells Grown in Culture

In order to enumerate cells after 8 days of culture, ten 20x images per well were taken (Leica inverted microscope DMI6000, Leica UK) in duplicate. The total area of a single well is covered by approximately eighteen of these 20x images and therefore we extrapolated the cell count to report total number of cells per well. Image J version 1.48c application *cell counter* was used to track cells during cell counting.

### Immunohistochemistry (IHC)

The tissue biopsies were embedded in paraffin wax and sectioned into 5 µm sections. We followed the usual immunohistochemistry procedure with antigen retrieval for 30 min in Dako antigen retrieval solution (Dako, UK) at 95°C. After washing with PBS tissue sections were incubated in blocking buffer (PBS+2% FBS) for half an hour and then incubated in primary mouse anti human CD34 and rabbit anti-human Collagen 1 (1∶100 dilution) antibodies (Abcam, UK) overnight at 4°C. Secondary anti-mouse FITC conjugated (1∶250 dilution) and anti-rabbit AF-546 conjugated (1∶250 dilution) antibodies (Jackson laboratories, US) were added to the sections after thorough washing with PBS and incubated for an hour at room temperature. DAPI was added to the tissue section for nuclei staining. Isotype matched antibodies were used as a negative control.

### Immunocytochemistry (ICC)

Fibrocytes cultured for 8 days were fixed for 1 h in 10% neutral buffered formalin (Sigma-Aldrich, UK). After washing with PBS cells were incubated in Odyssey blocking buffer (LI-COR Biotechnology, UK Ltd) for 30 min and then incubated in primary mouse anti human CD34, collagen 1 and LSP-1 (all 1∶200 diluted in PBS) antibodies (Abcam, UK) overnight at 4°C after washing with PBS. Cells were labelled by anti-mouse fluorescein isothiocyanate (FITC) conjugated (1∶250 dilution) and anti-rabbit AlexaFluor-546 conjugated (1∶250 dilution) antibodies (Jackson laboratories, US) after thorough washing with PBS and incubated for 1 h at room temperature. DAPI was added to stain nuclei.

### Statistical Analysis

FACS data was analyzed using GraphPad Prism version 5.00 for Windows (GraphPad Software, San Diego California USA, www.graphpad.com). An unpaired two-tailed t-test was performed to check the statistical significance of the results. Paired t-test was also performed to analyze differences between CT and DD cell count at each concentration of SAP.

## Results

### Identification of Fibrocytes in DD and CT Blood

**Figure 2:**
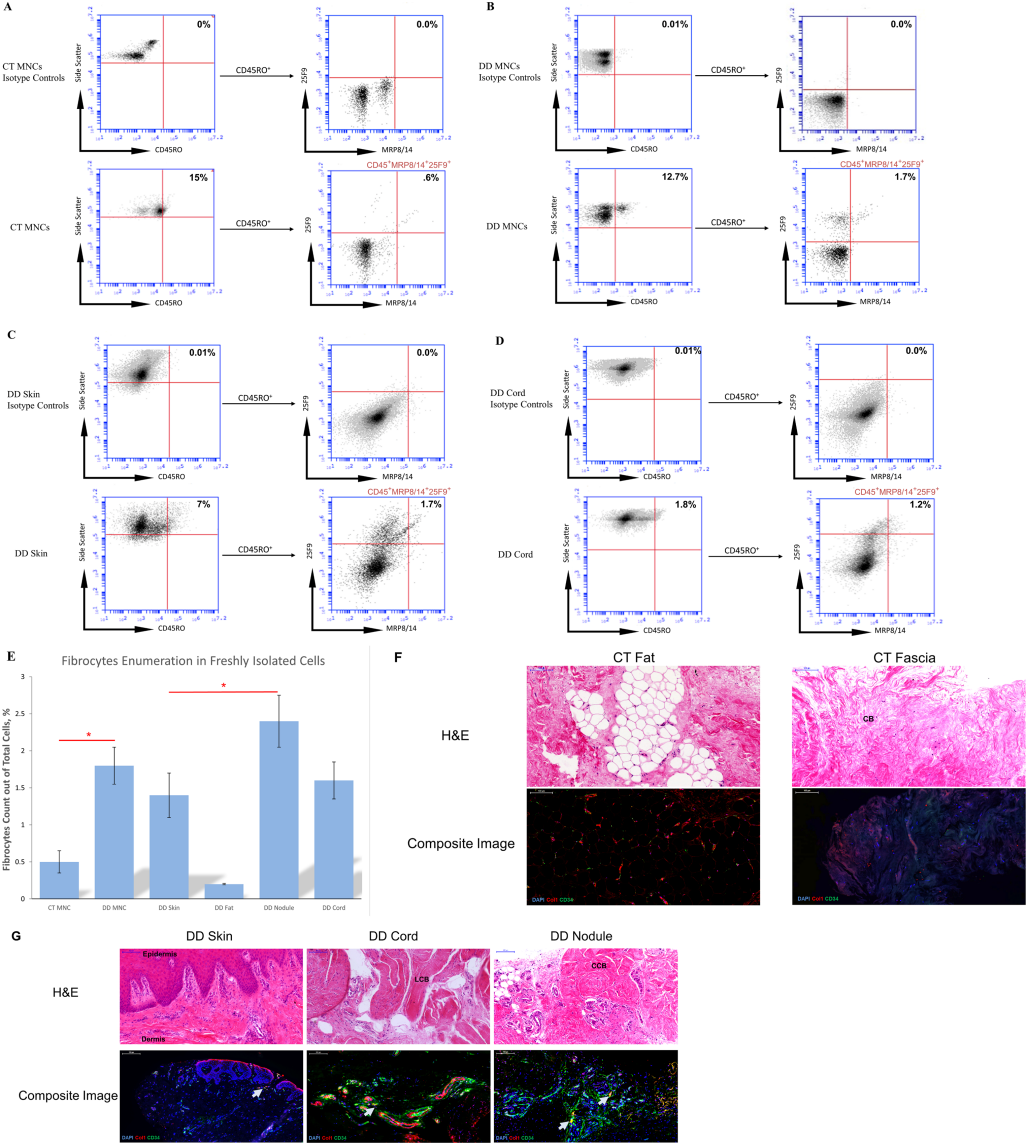
Presence of fibrocytes in dupuytren’s disease (DD) and control carpal tunnel (CT) blood and tissue confirmed through FACS and immunohistology. (**A–D**) The gating strategy used in identifying triple positive cells in CT and DD samples. The cells were labelled with antibodies against CD45RO, 25F9 and MRP8/14 cell surface markers. In a CD45RO vs. side scatter plot, CD45RO^+^ were selected and these cells were gated in a 25F9 vs. MRP8/14 plot. In the second plot, cells positive for both 25F9 and MRP8/14 were selected (along with total population as the gating was shown to be unnecessary as all 25F9^+^/MRP8/14^+^ cells were always CD45RO^+^) that are now considered to be triple positive (CD45RO^+^/25F9^+^/MRP8/14^+^). Cell viability was assessed separately through propidium iodide labelling. Cells were analyzed on Accuri C6 flow cytometer as explained in the materials and methods. (**E**) The fresh cells were obtained from the DD blood and tissue biopsies as explained in the methods. The cells were washed and passed through 70 µm nylon mesh filter before being labelled with antibodies as described above. Fibrocytes were highly expressed in freshly isolated MNCs from blood (1.8%) and in DD nodule (2.4%) compared to other tissue types. An unpaired two-tailed t-test was performed to check the statistical significance of the results. Error bars represent standard deviation. (**F**) H&E and immunofluorescence images of carpal tunnel (CT) fat and palmar fascia (n = 4 patients). CT fat and fascia are shown to be almost completely devoid of doubly positive collagen 1/CD34 cells. Red = Collagen I, Green = CD34, Blue = DAPI. CB = Collagen bundles. (**G**) H&E and immunofluorescence images of DD skin, cord and nodule (n = 4 patients). Cord typically shows linear arrangement of collagen bundles (LCB), while nodule here show circular arrangement of collagen fibers (CCB). DD cord and nodule showed the presence of clusters of doubly positive collagen 1/CD34 cells. Arrows points to the doubly positive cells that appeared as orange. Red = Collagen I, Green = CD34, Blue = DAPI, Orange = doubly positive cells. LCB = Linear collagen bundles; CCB = Circular collagen bundles. All scale bars: 100 µm.

In order to quantify the number of fibrocytes in mononuclear cells (MNCs) obtained from carpal tunnel (CT) subjects and DD patients’ blood (n = 10), we employed simultaneous labelling of three cell surface markers shown earlier to uniquely identify fibrocytes. These cells were then analyzed on a flow cytometer to assess the percentage of cells positive for CD45RO, 25F9 and MRP8/14. We found that DD blood contained significantly (p = 0.04) higher number of triple positive cells (1.8%) compared to control CT blood (0.5%) (Figure 2, A–B, E).

### Fibrocyte Identification in DD Biopsies

We then looked for the presence of fibrocytes in the freshly isolated cells obtained from cord, nodule, skin and fat of DD patients (n = 10). Interestingly, we found that nodule yielded significantly higher number of fibrocytes (2.4%) compared to other tissue types from the same patient (Figure 2, C–E). Cord came second with an average of 1.6% cells and skin followed with 1.4% of cells identified as fibrocytes out of total live cells obtained from the tissue biopsies. The least number of fibrocytes were detected in fat (0.2%), which was also used as a control tissue for each patient.

In order to verify the presence of fibrocytes *in situ,* we labelled control carpal tunnel fat and fascia tissue sections for collagen I/CD34 and compared them to DD skin, cord and nodule. DD cord and nodule tissues were shown to express doubly positive collagen I/CD34 cells, while control carpal tunnel tissues were devoid of these cells (Figure 2F & G).

### SAP Concentration in CT and DD Patients

**Figure 3:**
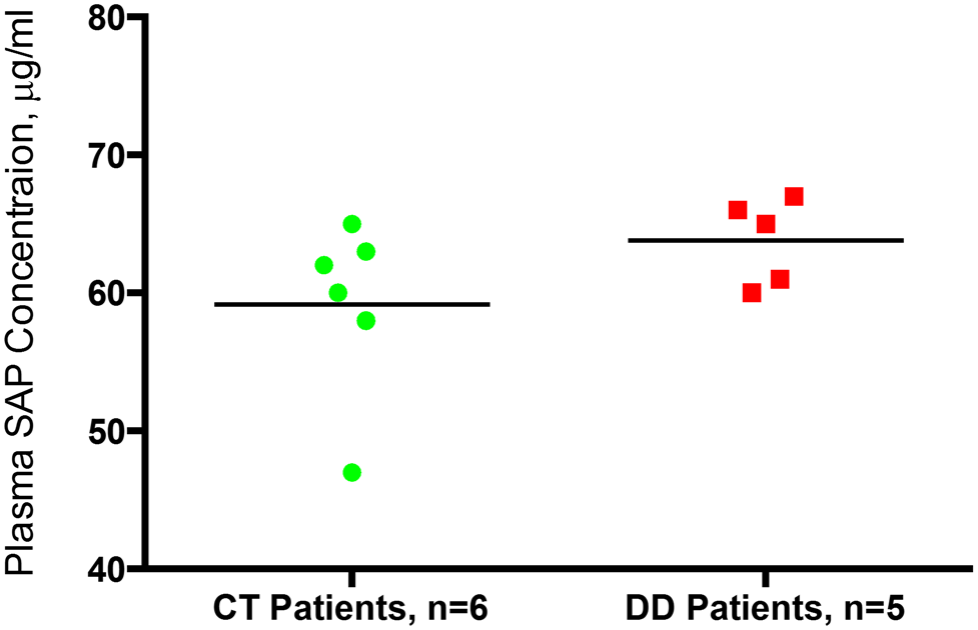
Determination of SAP levels in dupuytren’s disease (DD) cases and control carpal tunnel (CT) subjects’ blood. The plasma SAP level of DD (n = 5) and CT subjects (n = 6). No significant difference (*p* = 0.28) was found between the plasma SAP concentration of DD and CT patients. DD = Duputren’s Disease, CT = Carpal Tunnel, SAP = Serum Amyloid P.

The SAP concentration in CT control subjects and DD cases were detected and compared using sandwich ELISA in duplicates. The SAP concentrations detected in both CT (59.5±6.6 µg/ml) and DD (63.2±2.9 µg/ml) patients were generally higher compared to normal values (43±10 µg/ml, all male) as reported in the literature [33]. Although, we found no significant difference in plasma SAP concentrations between CT control compared to DD samples, p = 0.28 (Figure 3).

### Fibrocyte Culture and Enumeration

**Figure 4:**
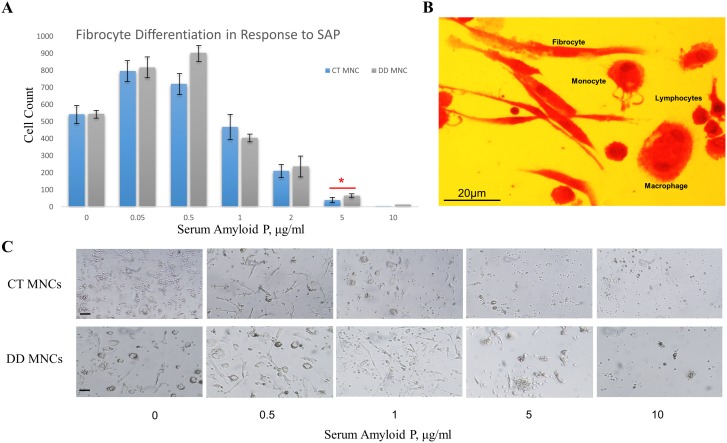
Blood derived fibrocyte differentiation in the presence of serum amyloid p (SAP). (**A**) Fibrocyte counts at day 8 of **dupuytren’s disease** (DD) subjects (n = 10 patients) were higher than control carpal tunnel (CT) (n = 10 patients) for all concentrations of SAP. No significant difference was observed between CT and DD fibrocyte inhibition by SAP up to 2 µg/ml of SAP concentration. At 5 µg/ml SAP concentration virtually all CT MNCs were inhibited to differentiate into fibrocytes (p = 0.03). It took 10 µg/ml SAP to significantly reduce the number of fibrocytes for DD cultures. Error bars represent standard deviation. (**B**) H&E staining of day 8 fibrocytes (n = 5 patients), magnification 40x. Alongside of fibrocytes various other blood cells are visible, including monocytes, lymphocytes and a macrophage. (**C**) The bright field images of fibrocyte cultures at various concentrations of SAP. Magnification bar = 50 µm.

Fibrocyte cultures were established from blood for DD (n = 10) and control CT (n = 10) samples. CT and DD samples showed no significant (p = 0.3) difference in fibrocyte count at the end of an 8 day period when grown without SAP. Initially, we applied SAP at increasing concentrations of 0.05, 0.5, 1, 2, 5, 10, 20 and 40 µg/ml to both CT and DD blood samples. Although, we found no appearance of fibrocytes at SAP concentrations of 20 and 40 µg/ml (data not shown) and therefore for all the following experiments, we used a 10 µg/ml SAP concentration as the upper limit. Interestingly, we found that compared to using no SAP; addition of 0.05 µg/ml of SAP resulted in a more robust growth of fibrocytes (Figure 4, A&C). We assessed the morphology of fibrocytes using H&E, which showed that the cells grown matched their morphological description with typical long spindle shaped cells with small central nucleus (Figure 4, B). No significant difference was observed in proliferation of fibrocytes form CT and DD MNCs up to 2 µg/ml SAP concentrations (Figure 4A). At a concentration of 5 µg/ml SAP CT MNCs derived cells were significantly inhibited by SAP compared to DD MNCs (p = 0.03). Due to the fact, that very few cells were observed at 10 µg/ml SAP for both CT and DD, we could not detect a statistical difference between the two groups.

**Figure 5:**
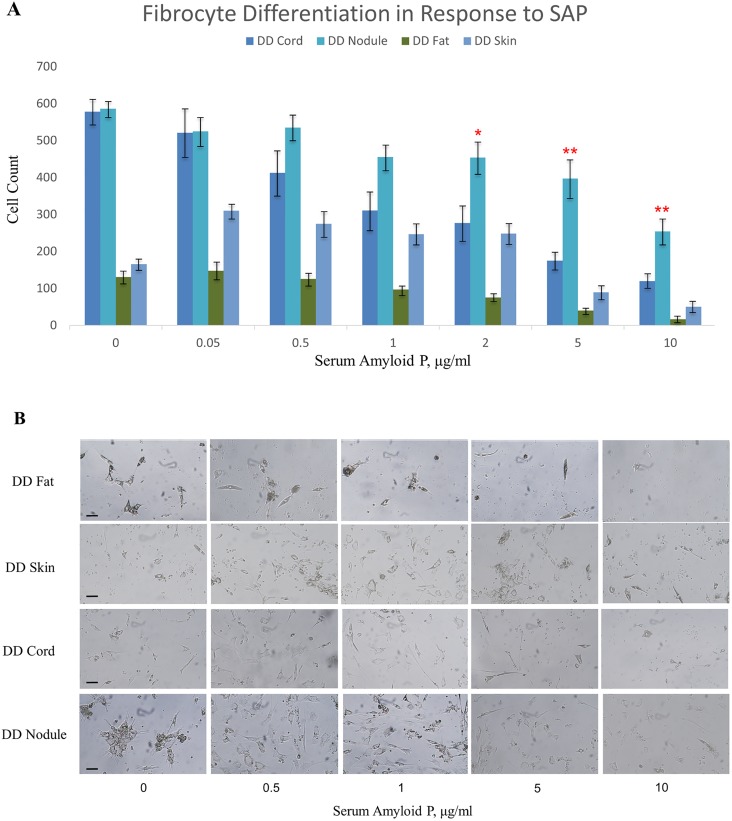
Differentiation of fibrocytes derived from Dupuytren’s disease (DD) biopsies . (**A**) DD nodule, cord, skin and fat derived cells were cultured (2×10^4^ cells in 200 µl serum free media) in 24-well plates for 8 days. 20x images were taken and cell count was performed as described in the materials and methods. Fibrocytes were shown to differentiate up to 10 µg/ml of serum amyloid P concentration, mainly from cord and nodule. Fat derived cells, used as control, showed the least growth. Nodule derived cells showed significantly higher potential to differentiate up to 10 µg/ml of serum amyloid P concentration. Paired t-test was performed to analyze differences between CT and DD cell count at each concentration of SAP. Error bars represent standard deviation. (**B**) The bright field images of fibrocyte cultures derived from DD tissue at various concentrations of SAP. Magnification bar = 50 µm.

In order to identify fibrocytes in DD tissue, we chose fat as control tissue against skin (mainly dermis), nodule and cord tissues from the same individual. However, we found that cell concentrations of 1×10^4^ cells per well in 200 µl of media was not adequate to yield sufficient number of fibrocytes to perform any analysis by day 8, this was especially true for fat cells which yielded very few fibrocytes. We therefore increased the number of initial cell density to 2×10^4^ cells per well, which proved to be sufficient for our final analysis. We found that both skin and fat tissues were inhibited by SAP in differentiating into fibrocytes. Although, we knew from our FACS data that there will be few of these cells. For the initial concentration of SAP, we found no significant difference between cord and nodule, but beyond 5 µg/ml SAP, only nodule showed a significant fibrocyte proliferation compared to cord and skin (Figure 5).

### Xiapex is More Effective in Fibrocyte Inhibition in DD Compared to Collagenase A

**Figure 6:**
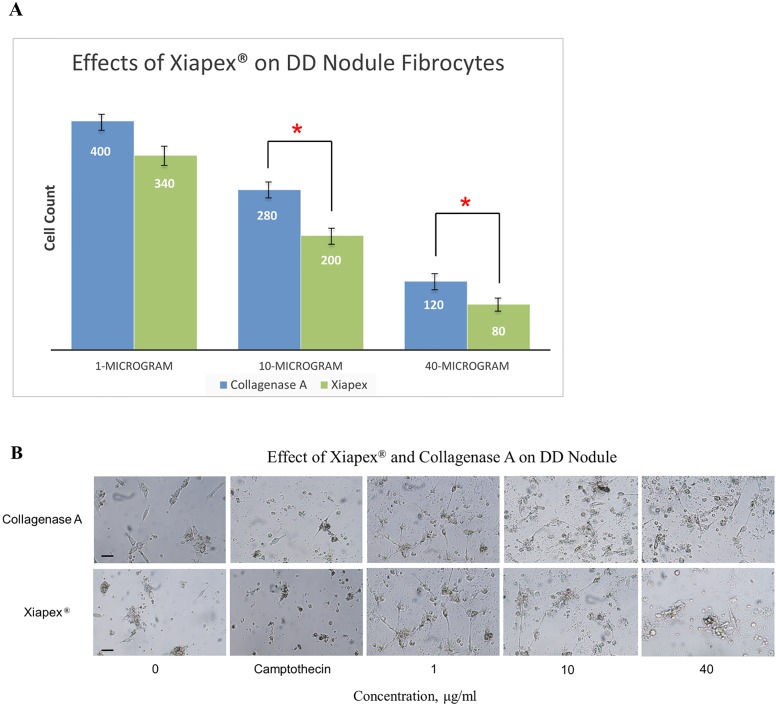
Effects of Xiapex on dupuytren’s disease (DD) nodule. (**A**) 2×10^4^ cells derived from nodule were grown in 200 µl serum free media and treated with increasing concentrations of Xiapex (Collagenase *Clostridium histolyticum*) and collagenase A. Cell count was performed at day 8 as described in materials and methods. At concentration of 10 µg/ml and higher Xiapex inhibited differentiation of fibrocytes significantly (p<0.05) compared to collagenase A. Error bars represent standard deviation. (**B**) The brightfield images of fibrocyte cultures derived from DD nodule at various concentrations of Xiapex and collagenase A. Magnification bar = 50 µm.

We set out to determine whether Xiapex (a special formulation of Collagenase *Clostridium histolyticum* by Auxilium Pharmaceuticals, USA) is more effective in inhibiting fibrocyte differentiation compared to commercially available collagenase A (Roche Diagnostics, UK). We initially used nanogram amounts of Xiapex but found it to be too low for having any effects on the fibrocyte culture (data not shown). When we administered between 1 and 40 µg/ml of Xiapex (based on dosages used in clinical practice) [34], we found that more than 10 µg/ml was useful in inhibiting fibrocyte differentiation compared to collagenase A (Figure 6). Even though, the inhibition was not complete, we were able to observe a 4 fold decrease in cell population compared to the control without Xiapex.

### Cells Grown as Fibrocytes were Shown to be Doubly Positive for Fibrocyte Specific Markers

**Figure 7:**
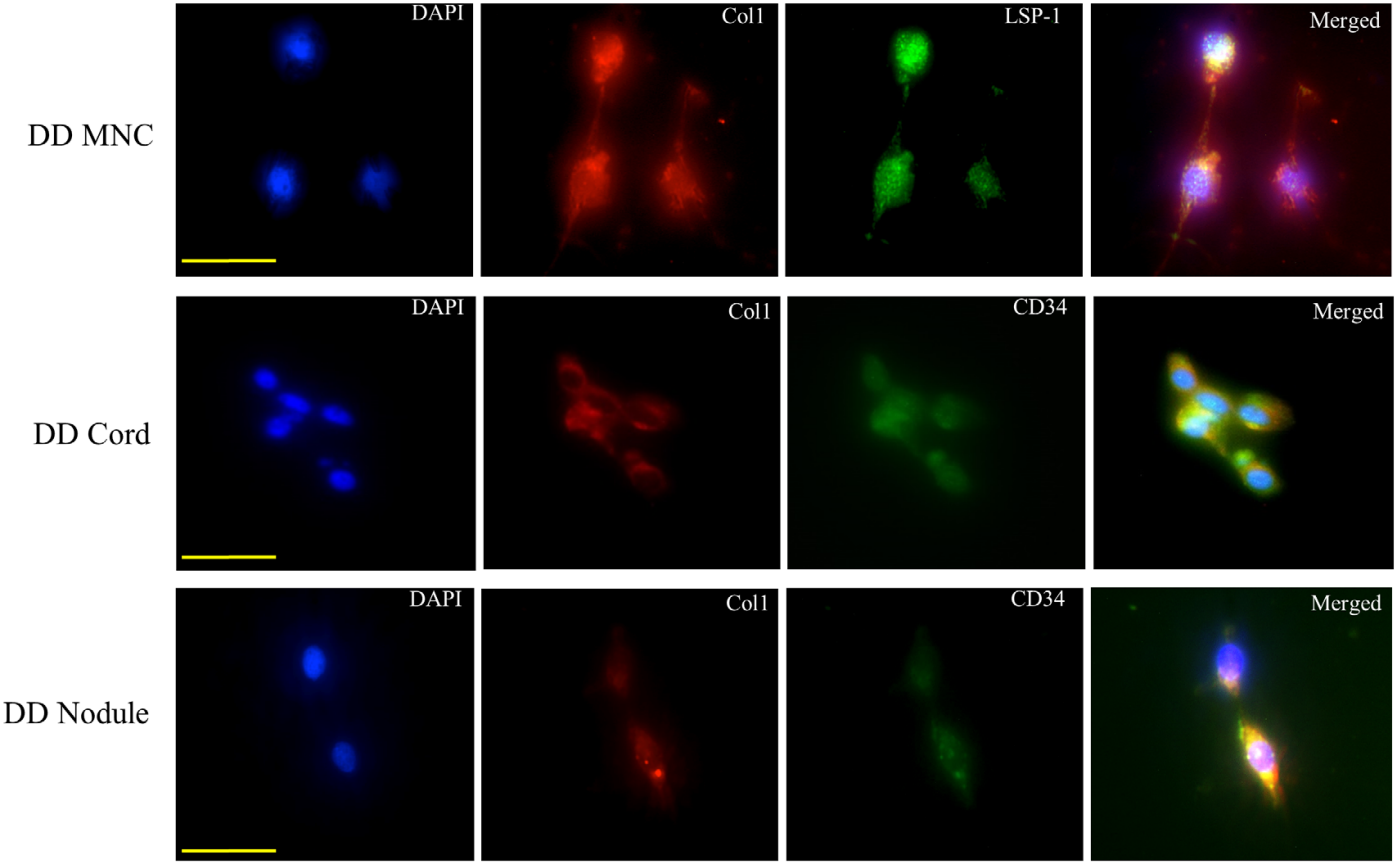
Immunofluorescent labelling of cultured fibrocytes (n = 6 patients). Fibrocytes have been found to be double positive for Col1/LSP-1 and Col1/CD34. Our labelling here shows that mononuclear cells (MNCs) from dupuytren’s disease (DD) blood and DD fascia with or without serum amyloid P treatment were positive for both set of markers. We therefore confirmed that the cells in our experiments were fibrocytes and not resident fibroblasts. Magnification bar = 20 µm.

In order to verify that the fibrocytes derived from blood and DD tissues were authentic fibrocytes; we showed that these cells were indeed doubly positive for Col1/CD34 (Figure 7) [17]. Furthermore, recent studies have shown that fibrocytes are also doubly positive for lymphocyte specific marker-1 (LSP-1) and collagen 1. We therefore looked for this particular marker in DD tissue and found that with or without SAP treatment, cells were positive for Col1/LSP-1 as well (Figure 7).

## Discussion

The findings of this study show for the first time the presence of fibrocytes in both DD tissue and blood. Nodular tissue from DD biopsies contained a significantly higher number of fibrocytes compared to cord and dermis. Moreover, the nodule derived fibrocytes were more resistant to SAP and Xiapex (CCH) compared to other tissue types. Fibrocytes have been shown to play an important role in wound healing and tissue regeneration. On the other hand, their malfunction and/or increased proliferation can lead to development of tissue fibrosis [35]–[37]. Dupuytren’s disease provides a useful fibrotic model for identification and evaluation of the role of fibrocytes in progression of tissue fibrosis [38], [39].

Since serum can inhibit fibrocyte proliferation, due to the presence of SAP, we chose to grow cells obtained from blood and tissue in serum free media [23], [40]. Without SAP treatment, cells obtained from both DD and CT blood showed no significant difference in their fibrocyte differentiation (Figure, 4A). However, serum has been shown to inhibit fibrocyte differentiation, of which SAP is the main inhibitory component [20], [23]. Thus, we chose to assess SAP concentration in both DD and CT patient’s blood. Our analysis showed that there is no significant difference in SAP concentration between DD and CT blood (Figure 3). Interestingly, 0.05 µg/ml treated SAP cultures, although not significant, compared to absence of SAP showed a noticeable growth. This effect has already been reported earlier where low SAP or low serum concentrations had shown to promote fibrocyte differentiation [41].

The fibrocytes from DD blood were more resistant to SAP inhibition compared to CT blood (Figure 4). The effect of SAP was not pronounced for reduced concentrations of up to 2 µg/ml, but the CT derived cells were inhibited significantly at 5 µg/ml SAP concentration. On the other hand, DD cells were completely inhibited by 10 µg/ml of SAP. Since, SAP concentration was similar in both DD and CT blood, we propose that there may be an inherent difference in the unique characteristics of fibrocytes present in DD compared to CT. Therefore, this difference could potentially be evaluated and targeted in the clinic setting when treatment or preventive therapy may reduce risk of recurrence of DD.

In view of the differences observed in the basal myofibroblast population in the cord and nodule [42], we set out to determine the differential concentration of fibrocyte populations in various subtypes of DD tissue. We first looked into the simultaneous expression of 3 known markers that have been shown to uniquely identify fibrocytes [29]. These markers were lymphocyte specific protein-1 (LSP-1) that is expressed only in lymphocytes, 25F9 and MRP8/14 that are expressed in only macrophages and few in monocytes [43]–[45]. Our results clearly showed that DD blood and DD tissue contain low percentage of fibrocytes among the total cells analyzed (Figures 2&5). We found that nodule contained significantly higher numbers of fibrocytes compared to other DD tissue types (2.4% nodule vs. 1.4% of skin). Previously, we had shown that the number of MSCs were higher in DD cord compared to nodule but however, in this study we have now shown the opposite finding in terms of fibrocyte expression [7]. This finding points to the possibility that the main progenitors are likely to be fibrocytes residing in nodules, which may gradually differentiate into mature myofibroblasts.

The SAP inhibition of DD tissue showed that DD nodule derived cells were significantly more resistant to SAP (Figure 5). We found that there was a disparity in the final count of cells at higher concentrations of SAP, at 5 and 10 µg/ml, which is not due to differences in inherent properties of fibrocytes, but in the starting numbers of fibrocyte precursors in DD blood and tissue. SAP regulation of fibrocyte numbers in DD is not possible as the serum proteins leakage into the fascia is likely to be minimal. Therefore, the nodular fibrocytes may form a niche of their own which can affect the local progression of DD through continuous differentiation into fibroblasts that ultimately may differentiate into myofibroblasts.

Additionally, we assessed the effect of Xiapex on fibrocytes derived from DD blood and tissue. Since Xiapex is a combination of 2 slightly different collagenases from *Clostridium histolyticum*, we also treated these cells with a commercially available collagenase A in order to compare its effect with that of Xiapex. The results showed that Xiapex is significantly more effective against fibrocyte differentiation from both MNCs and tissue derived cells (Figure 6). This inhibition was not apparent until we increased the concentration of Xiapex to 10 µg/ml. We had previously shown that Xiapex is effective in down regulation of ECM secretion in DD fibroblasts [32]. Our results therefore may imply that the combination of collagenases in Xiapex are more effective in inhibiting release of collagen by fibrocytes precursors, which may result in minimal contact with surface of these cells, a primary requisite for fibrocyte differentiation.

Importantly, we checked the identity of fibrocytes before and after any treatment using FACS. This was checked using double fluorescence for Col1/CD34 and Col1/LSP-2 for cells with or without drug treatments, considered to be a sufficient criterion to designate a cell as fibrocyte, (Figure 7) [29], [46]. It was important to show that the cells grown from DD tissue are representative of the canonical fibrocytes and not just any form of fibroblast. Therefore our analysis shows that the inhibition caused by SAP and Xiapex is against true fibrocytes and not against fibroblasts.

Since the numbers of fibrocytes were minimal per well; we were not able to further investigate the gene expression profiling for certain fibrotic markers of significance related to fibrosis and DD such as TGFβ. In future, it is envisaged that it will be helpful to develop means by which to relate and compare our data with gene expression profiling of important profibrotic markers pre and post drug inhibition. It would be relevant to establish whether inhibition caused by these drugs is specifically aimed at stopping fibrocyte precursors to secret profibrotic factors, or whether the observed effects are merely the indirect effects of reduced attachment to cell surface by SAP through an unknown mechanism, or by inhibition of collagen production by Xiapex.
