# Expression of Concern: First identification of resident and circulating fibrocytes in Dupuytren’s disease shown to be inhibited by serum amyloid P and Xiapex

**DOI:** 10.1371/journal.pone.0339113

**Published:** 2025-12-17

**Authors:** The PLOS One Editors

After this article [1] was published, concerns were raised regarding results presented in Fig 6 and the statistical analyses reported in this study.

Specifically:

- In Fig 6B, part of the Collagenase A 1 µg/ml panel appears similar to part of the Xiapex® 1 µg/ml panel
- The Statistical Analysis subsection of the Materials and Methods reports that data were analyzed using unpaired two-tailed t-tests, and paired t-test for differences between CT and DD cell counts at each concentration of SAP. However, multiple experiments in this article [1] appear to present more than two variables, suggesting that statistical tests designed for the comparison of multiple variables should have been used instead.

The corresponding author, AB, stated that the data underlying the results in [1] are no longer available, and the underlying data were not provided with the published article [1], contrary to the Data Availability statement. This article [1] therefore does not comply with the PLOS Data Availability policy.

The *PLOS One* Editors issue this Expression of Concern to inform readers of the above unresolved concerns.
